# Mechanical Stress Downregulates MHC Class I Expression on Human Cancer Cell Membrane

**DOI:** 10.1371/journal.pone.0111758

**Published:** 2014-12-26

**Authors:** Rosanna La Rocca, Rossana Tallerico, Almosawy Talib Hassan, Gobind Das, Lakshmikanth Tadepally, Marco Matteucci, Carlo Liberale, Maria Mesuraca, Domenica Scumaci, Francesco Gentile, Gheorghe Cojoc, Gerardo Perozziello, Antonio Ammendolia, Adriana Gallo, Klas Kärre, Giovanni Cuda, Patrizio Candeloro, Enzo Di Fabrizio, Ennio Carbone

**Affiliations:** 1 Department of Experimental and Clinical Medicine, University of “Magna Graecia”, Catanzaro, Italy; 2 Italian Institute of Technology (IIT), Genova, Italy; 3 Department of Microbiology, College of Medicine, University of Thi-Qar, Nasseriah, Iraq; 4 King Abdullah University of Science and Technology, Thuwal, Kingdom of Saudi Arabia; 5 Science for Life Laboratory, Department of Medicine, Karolinska Institute, Stockholm, Sweden; 6 Nanotech Department of Micro- and Nanotechnology, Technical University of Denmark, Kongens Lyngby, Denmark; 7 Department of Surgical and Medical Sciences, University of “Magna Graecia”, Catanzaro, Italy; 8 Endocrinology and Experimental Oncology Institute, CNR, Napoli, Italy; 9 Department of Microbiology, Tumor and Cell Biology, Karolinska Institute, Stockholm, Sweden; Dalhousie University, Canada

## Abstract

In our body, cells are continuously exposed to physical forces that can regulate different cell functions such as cell proliferation, differentiation and death. In this work, we employed two different strategies to mechanically stress cancer cells. The cancer and healthy cell populations were treated either with mechanical stress delivered by a micropump (fabricated by deep X-ray nanolithography) or by ultrasound wave stimuli. A specific down-regulation of Major Histocompatibility Complex (MHC) class I molecules expression on cancer cell membrane compared to different kinds of healthy cells (fibroblasts, macrophages, dendritic and lymphocyte cells) was observed, stimulating the cells with forces in the range of nano-newton, and pressures between 1 and 10 bar (1 bar = 100.000 Pascal), depending on the devices used. Moreover, Raman spectroscopy analysis, after mechanical treatment, in the range between 700–1800 cm^−1^, indicated a relative concentration variation of MHC class I. PCA analysis was also performed to distinguish control and stressed cells within different cell lines. These mechanical induced phenotypic changes increase the tumor immunogenicity, as revealed by the related increased susceptibility to Natural Killer (NK) cells cytotoxic recognition.

## Introduction

In nature, cells are continually exposed to physical, chemical and biological stresses. In the past, physical changes occurring in pathological tissues were taken into account by the physicians as valuable diagnostic indicators. Physical stress is involved in the pathophysiology of several human diseases, such as inflammation and cancer. In both conditions, an alteration in the chemical-physical extracellular matrix (ECM) environment is associated with the pathogenesis of these diseases. Moreover, physical forces play a significant role in metastatic progression. In recent years, novel tools, such as atomic force microscopy, have been developed to analyse changes in cells elasticity related to physical changes in the extracellular matrix compartment [1]. Furthermore, to determine how much a cell can be deformed, a device called “optical stretcher” was developed [2]. Unlike other tools, the optical stretcher is based on a double-beam optical trap [3], [4] in which two opponent and identical laser beams trap a cell in the middle. This method can be used to measure the elastic and contractile properties of many cells, as it is known that the cell's ability to contract is very important for migration and proliferation [5]. Furthermore, elasticity and contractility of different tumor cells may change with the progression of the disease, with an increased elasticity of the cancerous compared with the healthy cells [6]. A relationship between ECM stiffness and tumor transformation has been described [7]. It has been shown that ECM-mediated isometric forces are sensed by integrins, which regulate the phosphorylation of mechano-transducer kinases, such as ERK and Rho [8]. It has been also demonstrated that the increment of exogenous forces lead to an increased cell proliferation rate and induce tumor-like phenotypic changes. Finally, inflammatory breast cancer is known to exert a mechanical load due to the ECM changes, potentially leading to a higher metastatic potential [9].

On this basis, we hypothesized that mechanical stress could either affect the expression of cell antigens or induce the expression of stress-inducible molecules such as NKG2D receptor ligands [10] able to prime cytotoxic effector lymphocytes cell functions.

In the last years the discovery of immunoreceptors recognizing stress inducible proteins have broadened our knowledge on how the immune system is primed [11], [12]. These observations have fostered our interest in controlled stress delivery devices that could elicit a tumor immunogenic phenotype able to evoke an immune response, especially when the tumor has already been edited by cytotoxic lymphocytes [13].

Natural Killer cells are potent cytotoxic lymphocytes able to recognize freshly explanted cancer cells [14]–[16] and to spontaneously lyse certain tumor targets [17]–[19]. They are regulated by a delicate balance between inhibitory receptors, recognizing self MHC class I molecules, and activating receptors for stress-inducible molecules [20]. NK cells have the ability to identify and kill virally infected and malignant cells while sparing normal cells. The NK cells regulation was poorly understood until the late 1980's when the “missing self” hypothesis was proposed [21]. According to this hypothesis, down-regulation of MHC class I molecules during viral infection or malignant transformation triggers NK activation.

Here we ask whether the treatment of NK resistant cancer cells by mechanical stress could tip the balance between inhibitory and activating tumor expressing molecules in favour of the latter, leading to NK cell activation. In this work, we used two different procedures to mechanically stress cancer and normal cells under controlled conditions. We compared the biological effects of mechanical stimuli delivered either by a micropump device engineered expressly for this purpose [22], to the ones delivered by a shock waves pulse equipment. The variation in MHC class I molecules before and after mechanical stress was monitored both by means of Raman spectroscopy (in combination with principal component analysis (PCA)) and by means of cytotoxic measurements. The ultimate goal of our study was to understand if the applied mechanical forces could elicit and/or modulate relevant biological cell features, such as their immunogenicity. Moreover, we explored the possibility to use adoptively mechanical manipulations toswitch a tumor NK cell resistant phenotype into a susceptible one.

## Materials and Methods

### Micropump device

To deliver mechanical stress to tumor cell populations, we used a previously described micropump [22] (S1A Fig.) fabricated by means ofdeep X-ray lithography (DXRL) technique to specifically treat eukaryotic cells without destroying them. Three million cells/ml were mechanically stressed for 1 hour at 48 cycles and the strength of the maximum pressure applied was about 10 bars. This was evaluated by measuring the pump prevalence by using water as a fluid. Afterwards the cells were collected in a 15 mL tubes and were analysed by flow cytometry and micro Raman spectroscopy.

### Shock Waves device

Shock waves are applied in orthopaedics and are essentially acoustic waves with a mechanical effect. When the shock waves pass through a fluid, create differences in pressure responsible for the formation of gas bubbles (cavitation) that are affected by subsequent shock waves and are deformed until the implosion. Asymmetric collapse of the bubble causes the formation of a jet of water “jet stream”. This phenomenon further enhances the mechanical effect of the shock wave causing micro-lesions whose size is a function of the number of pulses and flux of energy. For the treatment of tumor cell lines was used the *Swiss Dolorclast device* (Electro Medical System-EMS, Italy) with a hand-piece high-energy (S1B Fig.).

Cancer cells were grown in Petri dishes and treated with shock waves (500 shots) applying a flux of energy of 1 bar. After treatment the cells were collected and analysed, and the data obtained were compared to respective controls.

### Cells isolation and culture

For mechanical stress experiments we used different kinds of cell lines. Primary cells Mel 59c, Mel 42a, Mel 42b, Mel 66b, Mel 137a and Mel 103b were obtained from freshly explanted melanoma skin metastatic cells with few in vitro passages, respectively from patients 59, 42, 66, 137 and 103. All patients gave written informed consent according to the Declaration of Helsinki and the Ethics Committee II of Heidelberg University (0198.3/2002). Healthy donors PBL and related purified NK cells were generated accordingly with the Ethics Committee of University Magna Graecia of Catanzaro (49/2003). The study was approved by the UMG Ethics Committee (49/2003). Human kidney carcinoma cell line (293T), human B lymphoblastoid cell line (IM9), and fibroblast cells were obtained from the American Type Culture Collection (ATCC). Peripheral Blood Lymphocytes (PBLs) were obtained from healthy donors by Ficoll-Paque (Biochrom AG, Berlin, Germany) density gradient centrifugation the human samples were collected accordingly with the Ethics Committee of University Magna Graecia of Catanzaro (49/2003). Cells were grown in RPMI 1640 medium and in Dulbecco's Modified Eagle's medium (DMEM) supplemented with 10% of fetal bovine serum (FBS) (SIGMA Aldrich, St. Louis) and penicillin (100 IU/mL) and streptomycin (100 mg/mL) (SIGMA Aldrich, St. Louis) and were maintained at 37°C in a humidified 5% CO_2_ atmosphere. Macrophages and dendritic cells (DC) were obtained from blood mononuclear cells. Macrophages were generated from monocytes adherent on the plate. Dendritic cells (DC) were established from monocytes supplemented for 7 days with 50 ng/ml granulocyte/macrophage colony-stimulating factor (GM-CSF) (Santa Cruz Biotechnology, Texas, U.S.A.) and 1000 U/ml IL-4 [23].

### Natural Killer cell purification

NK cells were obtained as described [24]. Briefly, human NK lymphocytes were purified from peripheral blood mononuclear cells (PBMC), obtained from healthy donors by Ficoll-Paque density gradient centrifugation, using the NK Cell Isolation kit and Vario MACS for the depletion of non-NK cells (Miltenyi Biotec). Cells were resuspended in RPMI 1640 medium supplemented with 10% FBS, 3% human serum, penicillin (100 IU/ml), and streptomycin (100 µg/ml) and were used for cytotoxicity assay. NK cell purity was at least 95%.

### Raman Spectroscopy

Microprobe Raman spectra were excited by near IR laser with 830 nm laser line at room temperature (RT) in backscattering geometry through a 100X objective (*NA* = 0.95) of Leica microscope (Model - DMLM). All data were collected with a laser power of 70–130 mW. The laser power was always chosen in such a way to avoid any damage of cell. This laser power is not expected to cause any significant increase in sample temperature due to the extremely low absorption coefficient of cells at 830 nm. In addition, the cell conditions were verified through optical image before and after the measurements. Several cells of each cell line were probed in the range of 700–1800 cm^−1^ by point mapping measurements, which collect various spectra at different location covering the entire cell surface. For each cell line 5 cells were randomly chosen for point mapping. Raman spectra of different cells of each cell lines were then collected and used for statistical analysis (Spectra don't show significant different to each other [25], [26]). The statistical analysis (with standard deviation error) was performed for 10 Raman spectra for each cell line. It is noteworthy to point it out that the measurements for control and stressed cells of each cell line were performed on the same day to avoid any variation in instrumental response. Spectral smoothing was performed for all individual Raman spectra using the WiRE 2.0 PS9 software provided with the Renishaw Raman spectrometer. The curve fitting of the spectra were carried out using combined Gaussian and Lorentzian function.

### Principal Component Analysis

In the last decade multivariate techniques have been widely employed for analysis of large Raman datasets [27], [28]. Among these techniques, principal component analysis (PCA) has proven to be one of the most robust and reliable methods [29], [30]. When PCA is applied to Raman spectra, each Raman frequency is considered as a variable whose value changes across the recorded spectra, so that the whole dataset constitutes an *N*-dimensional space where *N* is the number of measured frequencies (*N* is typically very large). The main aim of PCA is dimensionality-reduction without loss of information. This is achieved through the diagonalization of the covariance matrix of spectra: the eigenvectors define one-dimensional (1D) axes in the *N*-space and the corresponding eigenvalues express the amount of total variance explained by each eigenvector. The eigenvectors are the so-called principal components (PCs), while the eigenvalues are named latents. The first principal component is 1D axis along which the largest amount of variance is taken into account (i.e. the axis with the largest latent value); the second principal component is the axis (orthogonal to the first one), which accounts for the largest residual variance (i.e. the axis with the second largest latent), and so on for the following components. In this way, from a *N*-variables dataset we are reduced to a few-variables dataset, without losing the significant part of information. PCA analysis was performed using the software developed by P. Candeloro et al. [31].

### Immunofluorescence assay

After each treatment, cells were collected in tubes and were incubated with human serum for 15 minutes at RT. Then, cells were washed three times in PBS 1X and subsequently incubated for 30 minutes in dark at 4°C, with the following mAbs: W6/32 (anti-MHC class I, IgG2a; BioLegend, San Diego, CA); clone BAM 195 (anti-MICA, IgG1) and mAb 6D4 (anti-MICA/B, IgG1), were kindly provided by Veronika Groh (Fred Hutchinson Cancer Research Center, Seattle, WA); M295 (anti-ULBP1, IgG1), M310 (anti-ULBP2, IgG1), M550 (anti-ULBP3, IgG3) and M478 (anti-ULBP4, IgG1) were kindly gifted by D. Cosman, (Amgen Inc. Seattle, WA); mAb L95 (anti-PVR, IgG1) and mAb L14 (anti-Nectin-2, IgG2a) kindly provided by A. Moretta (University of Genoa, Genoa, Italy). After washing twice, cells were stained with the secondary mAbs FITC goat anti-mouse IgG (Jackson Immuno Research, Baltimore, USA) for 30 min at 4°C in the dark. Then, cells were again washed twice and analysed by FACSCalibur flow cytometry (BD Bioscience, San Diego, CA, USA). For intracellular staining, cells were fixed in Cytofix and permeabilized by Cytoperm (BD Biosciences). The cells were stained with the specific mAbs followed by FITC conjugated anti-mouse IgG antibodies (Jackson Immuno Research, Baltimore, USA) and were analysed. The results were analyzed using Cell Quest (BD Biosciences) or FlowJo software version 9.3.1.

### Cytotoxicity assay

For the cytotoxicity assays the tumor or healthy cells as target and the NK lymphocytes as effector cells were used. After mechanical stress treatment, the target cells were incubated with the fluorescent CFDA (carboxyfluorescein diacetate, Life Technologies, NY, USA) for 30 min at 37°C, then washed twice and incubated with NK cells for three hours in incubator at 37°C and 5% CO_2_. The details of the protocol were described from McGinnes et al [32]. The flow cytometer was used to analyze the samples.

### Real Time

Total cellular RNAs from four different melanoma primary cells and two healthy cells were isolated using Trizol (Life Technologies).

Briefly, 1 µg of total RNA was reverse transcribed using the Superscript III Reverse Transcript Kit (Life Technologies) according to the manufacturer's recommendations; the cDNAs were amplified using iTaq Universal SYBER Green Supermix (BioRad, Segrate (MI), Italy) in the presence of specific primers as described previously [33]. The following primers were used: MHC-I- FW, 5′- CCTTGTGTGGGACTGAGAGG -3; MHC-I- RV, 5′- CAGAGATGGAGACACCTCAGC -3′. The expression of housekeeping gene glyceraldehyde 3-phosphate dehydrogenase (GAPDH) was used to normalize samples, and the relative quantification of HLA class I was performed applying the 2^−ΔΔCt^ method [34]. The following primers were used: GAPDH-FW, 5′-CACCATCTTCCAGGAGCGAG-3′, GAPDH-RV 5′-TCACGCCACAGTTTCCCGGA -3′.

### Western Blotting

The secretion of MHC class I molecules into the conditioned medium was confirmed by Western blot analysis. For Western blotting, cells cultured in 100 cm dishes were washed with 0.1 M phosphate-buffered saline (pH 7.4) (Life Technologies) and, thereafter, 2 mL of serum-free RPMI was added. After stress treatment, the medium was harvested. Cells medium proteins concentration for each stressed sample was measured in triplicate using the dye-binding protein (Bio-Rad) with human serum albumin (Life Technologies) as standard curve.

For each experimental point, 50 µg of cell-culture medium proteins were transferred to sterile tubes containing cold acetone (20%, w/v) and precipitated for 30 min on ice followed by centrifugation at 15000 rpm for 15 min at 4°C.

The supernatant was decanted, and the pellet washed with chilled acetone followed by removal of all the acetone. The pellet was subsequently solubilized in LB [31.25 mmol/L Tris-HCl (pH 6.8), 1.25% SDS, 6.25% glycerol, 0.06% bromophenol blue, and 5% h-mercaptoethanol], resolved by SDS-PAGE, and transferred to nitrocellulose membranes.

After addition of the blocking mixture [5% (w/v) milk in PBS (pH 7.4) and 0.05%Tween 20], the membrane was incubated with a 1∶100 dilution of mouse anti-HLA antibody, clone W6/32 (BioLegend,). The signal was detected with anti-mouse horseradish peroxidase-conjugated secondary antibody (1∶5000; Santa Cruz Biotechnology). The membrane was developed by enhanced chemiluminescence-Western blot detection reagents according to the manufacturer's instructions (Santa Cruz Biotechnology).

The membrane was incubated with Ponceau S red staining solution (Sigma-Aldrich) to ensure uniform gel loading. An internal control was not used because it is basically impossible to find a “housekeeping” protein in the serum free medium cells that could be used as a constant reference. Ponceau S can be used advantageously over actin detection for quality or equal loading control in Western blotting; moreover, it has an additional advantage, i.e. that it does not rely on a single protein for normalization or loading control. This circumvents the possibility that the “housekeeping” proteins used for this purpose may actually vary in some conditions or that they are saturated at the levels of loading necessary for detection of low-expression products or that they are not detectable as in our example [35].

### ATM/ATR signaling cascade analysis

Etoposide (Sigma, St. Louis, MO, USA) was dissolved in DMSO and added at the final concentration 5 µM for 1 h. Whole cell lysates were prepared from freshly collected cells by using a lysis buffer (20 mM Tris-HCl pH 7.5, 10 mM EDTA, 0,5% Nonidet P-40, 400 mM NaCl) supplemented with protease inhibitor mixture (Calbiochem, Merck Darmstadt, Germany), 0.5 mM PMSF and 2 mM sodium orthovanadate. Lysates were incubated on ice for 15 minutes and centrifuged at 13000 rpm for 10 min. Protein concentration from supernatants was determined by Biorad assay (Bio-Rad Laboratories, CA, USA). For immunoblots, samples were loaded in Laemmli buffer on 6% or 10% Tris-glycine SDS/PAGE gels, transferred to nitrocellulose membranes and hybridized with appropriate antibodies at 1∶1000 dilution. Blots were developed by enhanced chemiluminescence (Roche Diagnostic GmbH, Mannheim, Germany). Antibodies: mouse anti-phosphoAtm (Ser 1981) rabbit anti-phosphoChk1 (Ser 345), rabbit anti-phosphoChk2 (Thr 387), rabbit anti-phospho JNK (Thr183/Tyr185) (Cell Signaling, New York, NY, USA), rabbit anti -p53 and mouse anti-MCM7 (Santa Cruz).

### Statistical analysis

All results were reported as mean ± SEM. Significance level was determined by Mann – Whitney test. A value *p≤0.05, **p≤0,001 and ****p≤0,0001 was considered statistically significant. Data were expressed as fold change respect to the control, set as 1.

## Results

To understand the potential effects of mechanical stress on cell immunogenicity, cancer and healthy cells were mechanically stressed with a micropump device and shock waves.

**Figure 1:**
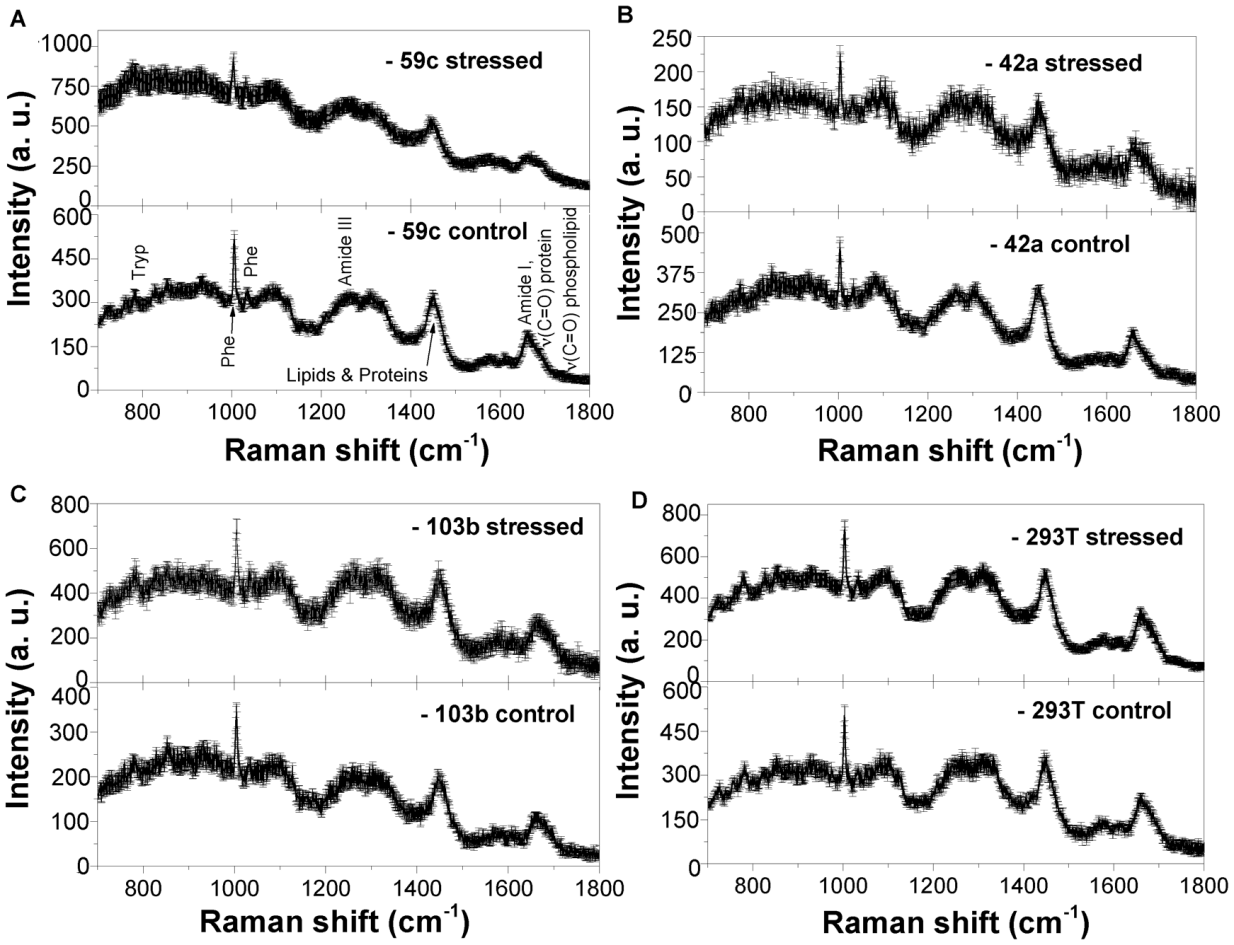
Raman spectroscopy of different cell lines. Raman spectra for control and stressed cancer cell lines (Mel 59c (A), Mel 42a (B), Mel 103b(C) and 293T(D)) with standard deviation error bar. The spectra were performed before and after mechanical stress with micropump.

The changes induced by the micropump-delivered stress were analysed by Raman spectroscopy. Raman measurements were performed for the respective cells (Mel 59c, Mel 42a, Mel 103a and 293 T cell line) in PBS solution in the spectral range between 700–1800 cm^−1^. Raman spectra with standard deviation error bar for control (unstressed) cells and mechanically stressed cells in PBS buffer solution are shown in Fig. 1. Raman spectra of these cells are similar to characteristic Raman spectra of most living cells. All the spectra show different peaks at about 780, 850, 1003, 1125, 1445, and 1660 cm^−1^. Typical bands in these spectra (Fig. 1) are associated to the nucleotide conformation (600–800 cm^−1^), molecular skeleton geometry and phosphate-related vibrations (800–1200 cm^−1^), nucleotides (1200–1600 cm^−1^), and C-C and C-H_x_ modes due to proteins and lipids [36]. The amide vibrations, such as the amide I-band (due to C = O stretching, 1650–1700 cm^−1^) and amide III band (due to C-N stretching, and N-H bending, about 1250 cm^−1^) in proteins are easily distinguishable [37]. The amide I bands in the range between 1650–1700 cm^−1^ give also very important information about the confirmation status of secondary structure (α-helix, β-sheet and random coil structure) of proteins. Different amino acids can be recognized explicitly at about 1003, 1011 and 1032 cm^−1^ in the Raman spectra [37].

**Figure 2:**
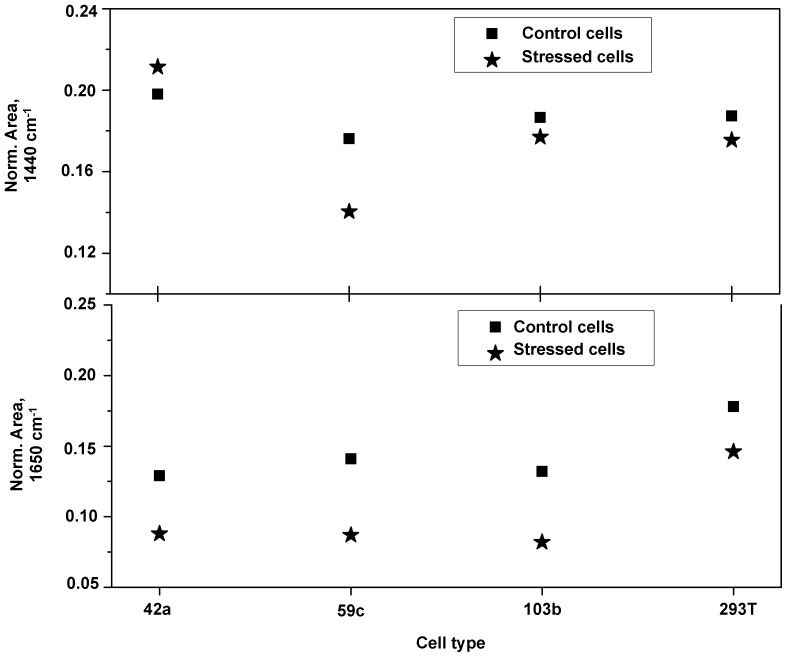
Protein concentration in different cell lines. Normalized area of band centred at around 1440 (top) and 1670 cm^−1^ (down) for all the cell lines, showing the variation of protein concentration and relative α-helix content over the cell surface.

The variation of relative concentration of protein in different cells was calculated by using curve fitting these two regions of protein bands at about 1440 and 1650 cm^−1^. Fig. 2 shows, for various cell types, the variation in relative intensity of cell membrane protein/lipid (1440 cm^−1^) and α-helix (1650 cm^−1^) band before and after the application of mechanical stress. A clear decrease of protein quantity and its α-helix secondary structure was observed upon mechanical stress cell treatment.

A very clear picture can be revealed from the variation of secondary structure after employing mechanical stress. It shows the reduction of α-helix secondary structure of amide I band for all the cells with mechanical stress. This trend is not clearly observed in Mel 42a cells for which the spectrum is very noisy (Fig. 1B). Furthermore, on deep analysis, it is found that the Mel 59c and 293 T cells behave in similar way. All the investigated cell lines show a diminishing secondary structure on mechanical stress. Quenching of α-helix structure and an enhancement of fluorescence background as well can be clearly observed. These alterations in the Raman spectra are consistent with our previously reported findings on the MHC class I detection by Raman spectroscopy [38].

**Figure 3:**
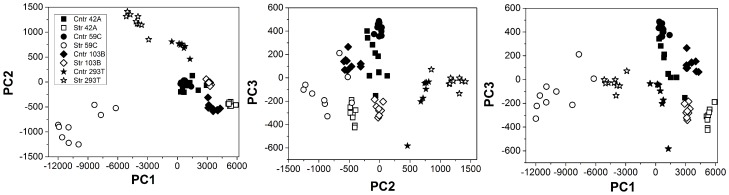
Principal component analysis. PCA analysis on control and stress cells for various cell lines; Mel 42a, Mel 59c, Mel 103b and 293T. a) PC1 vs. PC2, b) PC2 vs. PC3 and c) PC1 vs. PC3.

Furthermore, Principal Component Analysis (PCA) is performed to discriminate the stressed to control cells of each cell lines. PCA, a statistical analysis, reduces the dimensionality of multi-dimensional data set, keeping the characteristic of all the spectra [31]. The reduced dimension points of each spectrum is described by a limited number of variables, called principal components (PCs). These PCs incorporate most of the spectral information. Fig. 3 represents the PCA analysis (PC1 vs. PC2, PC2 vs. PC3 and PC1 vs. PC3) of all the cell lines in the range of 700–1800 cm^−1^. The filled blocks are for control cells whereas the empty blocks are associated to stress cells. The stress cells can be well distinguished from control cells and further each cell lines can be found in-grouped clearly in the Figure. These results reveal an interesting and important way to distinguish between cells and also if the cells have been perturbed externally.

To confirm that the mechanical stress induces a down-regulation of the MHC class I on the cells surface, we performed an immunophenotype assay for all the different cell types.

**Figure 4:**
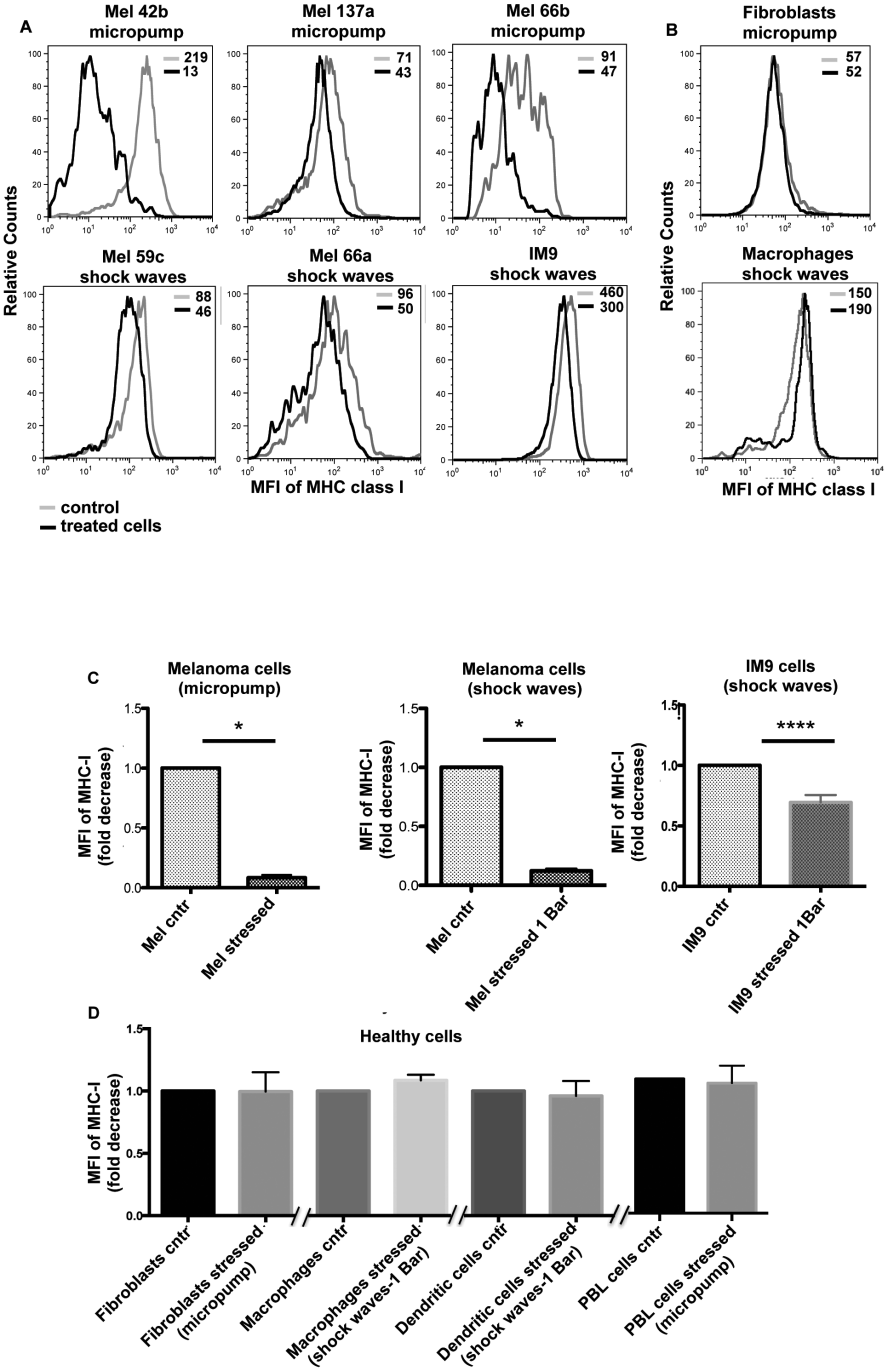
Median Fluorescence Intensity (MFI) of MHC-I, before and after mechanical stress. Panel A shows the decrease of the MHC class I molecules expression on cancer cells (5 melanoma and one lymphoblastoid cell lines) after mechanical stress treatment by means of the micropump (upper part) and the shock waves (lower part) compared with untreated cells. Panel B reports the effect of mechanical stress on some healthy cells (fibroblasts and macrophages) of the MHC class I with the two mentioned treatments. The related isotopic control MFI gave the same overlap signal for all cell lines used, therefore have been omitted. Panel C represents the statistical values of fold decrease of the MHC-I performed on melanoma cells (n = 4 separate experiment with micropump and n = 4 separate experiment with shock waves; p<0.05) and IM9 cells (n = 10 separate experiment with shock waves; p<0.0001). The fold decrease of MHC-I was derived from the Median Fluorescence Intensity (MFI) of MHC-I molecules before and after treatment of Melanoma and IM9 cell lines. The panel D shows the statistical values obtained from different experiment (n = 3) for each type of healthy cells. The fibroblasts and the PBLs were stressed with the micropump while, the macrophages and dendritic cells were treated with shock waves. The value reported in panel D is not statistically significant.

After a 1 bar power treatment, by micropump and shock waves, a clear reduction of MHC class I levels on the tumor cells membrane was observed (Fig. 4A), while no changes were observed when healthy cells, fibroblast, macrophage, dendritic and lymphocytes cells, were stressed (Fig. 4B). Statistical analyses were performed on tumor cells (melanoma and IM9 cell lines, fig. 4C) and healthy cells (fibroblast, macrophage, dendritic and lymphocytes cells, fig. 4D).

**Figure 5:**
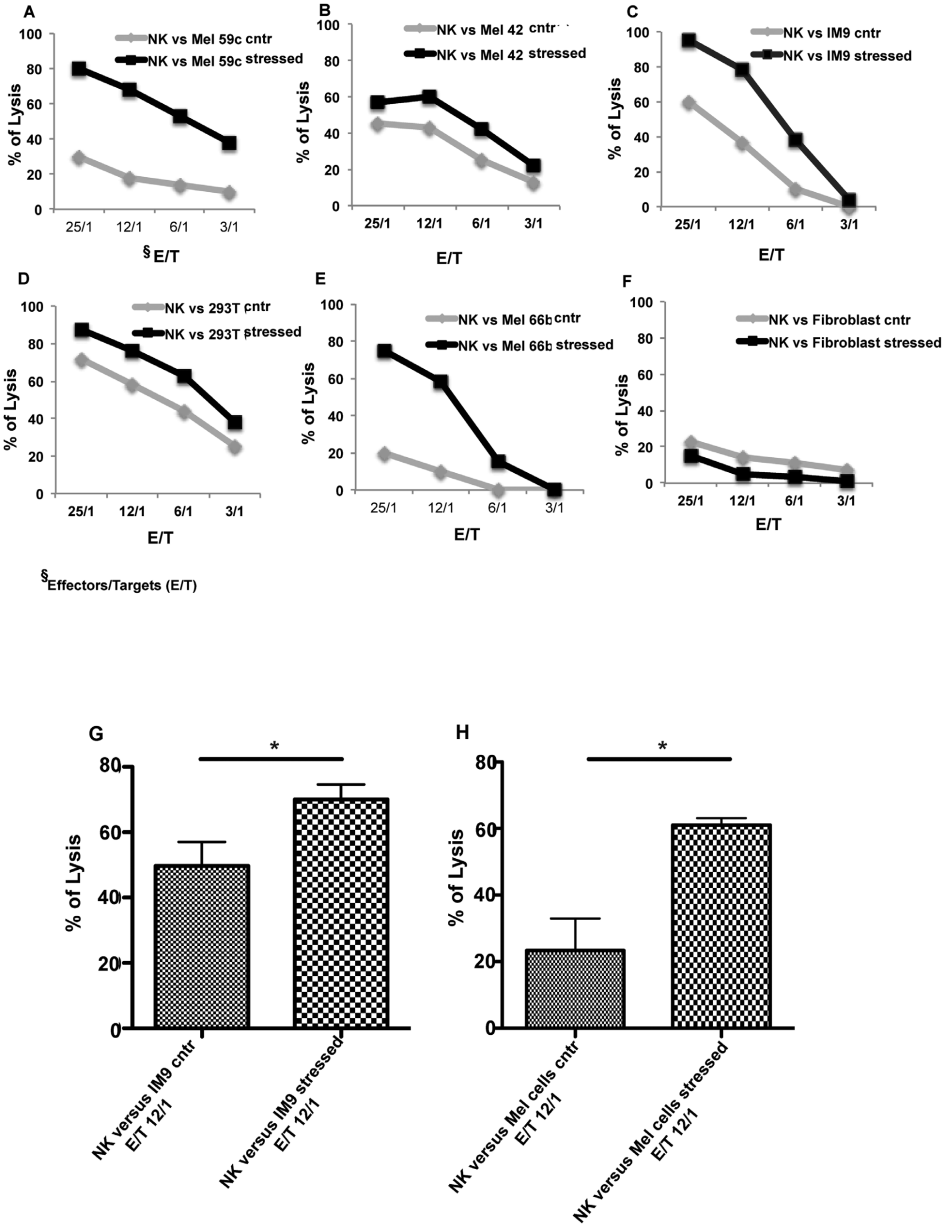
Increased NK susceptibility on mechanical stressed tumor cells. NK cell recognition of different tumor cell targets at different E/T (effector/target) ratio: 59c, 42a, 66b (melanoma cell lines), 293T (kidney carcinoma) and IM9 (lymphoblastoidcell lines) before (grey) and after (black) mechanical stress. The Mel 42a, Mel 66b, fibroblasts cells (panels B, E, and F) were treated with the micropump, the Mel 59c, IM9, 293 T cells (panels A, C and D) were stressed with the shock waves. As healthy target cells, in this case fibroblasts are shown. Representative experiments are reported for each cell type. Panels G and H show the statistics derived from three different functional assays, using NK lymphocytes as effectors cells (E) and IM9 and Melanoma cells as targets (T). The IM9 target cells were treated with the shock waves (panel G: n = 3, p = 0.0325), while the Melanoma target cells were stressed with the micropump (panel H, n = 3, p = 0.0186). E/T ratio 12/1, p<0.05.

The other immunogenic molecules analysed, such as MICA, MICB, ULBPs, PVR and Nectin-2, did not show significant changes between control and stressed cells with shock waves (S2 Fig.). To understand the effect of the decreased MHC class I expression on mechanically stressed tumor cells immunogenicity, functional assays were performed using both devices, micropump and shock waves. Herein, the NK cells susceptibility of mechanically stressed tumor target cells was compared with their unstressed controls by classic cytotoxicity assays. A clear and reproducible increase in the NK susceptibility was observed after mechanical stress treatment. The range of increasing NK lysis percentage on tumor cells was between 30–70% (Fig. 5A-E), while healthy cells, i.e. fibroblast (Fig. 5F), did not respond to mechanical stress treatment. The results show that mechanical stress improves the NK recognition for tumor with statistical significance (Fig. 5G-H), but not for healthy cells. Mechanical stress switches the tumor phenotype from being NK resistant to NK susceptible. This change in NK susceptibility correlates with tumor specific MHC-class I loss. The MHC class I molecules are the most potent inhibitory ligands for NK receptors. The MHC class I down-regulation on tumor cells trigger the NK response accordingly with the “Missing self hypothesis” [21]. The data here collected indicate that a shedding of MHC-I occurs after mechanical stress from tumor cell surface, this is not the case for healthy cells. Our finding indicates an immunologically relevant effect of mechanical stress on the tumor susceptibility to cytotoxic attack.

The increased cell cytotoxicity observed in classical NK cytotoxicity assays was not due by passive target cell death induced by mechanical stress treatments, but rather by active NK cells cytolitic program as witnessed by the reduction of mechanical stress target cells killing after NK cell's activating receptors blockade.

Next, we investigated the mechanism by which the mechanical stress, induced either with the micropump that with shock waves, may lead to MHC class I down regulation.

**Figure 6:**
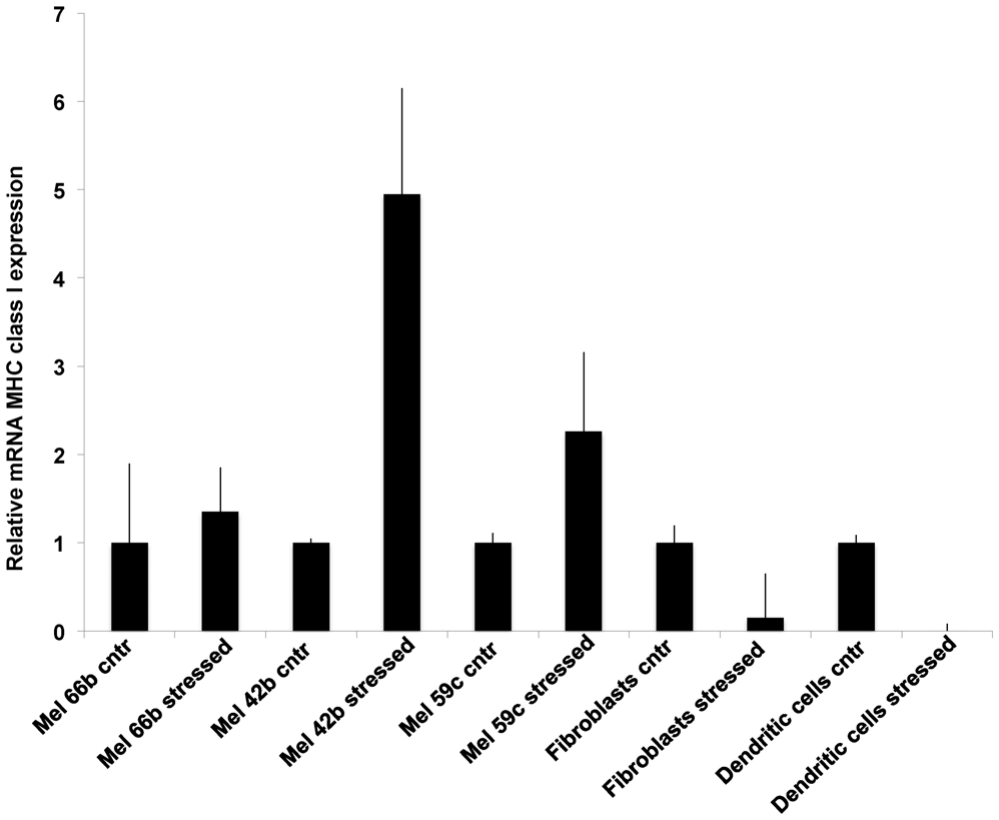
Fold increase of MHC-I mRNA from cancer and healthy cells after mechanical stress: Relative expression level of the MHC class I molecule was measured by qPCR on cells before and after applying micropump mechanical stress. Expression changes were related to unstressed samples. The results shown are the average of duplicates from 2 independent experiments. Error bars indicate standard deviations.

Fig. 6 shows the quantification of MHC class I mRNA levels in treated cells, compared with controls. The data demonstrate that, in melanoma cells, there is an increased level of transcripts after treatment, compared to the unstressed cells. We hypothesize that the reduction of MHC-I protein on treated cells surface might be followed by a reactivation of MHC-I gene transcription in order to restore the protein levels on cell membrane. Moreover, these data strongly suggest that the mechanical regulation of membrane associated MHC class I occurs at a post-transcriptional level.

**Figure 7:**
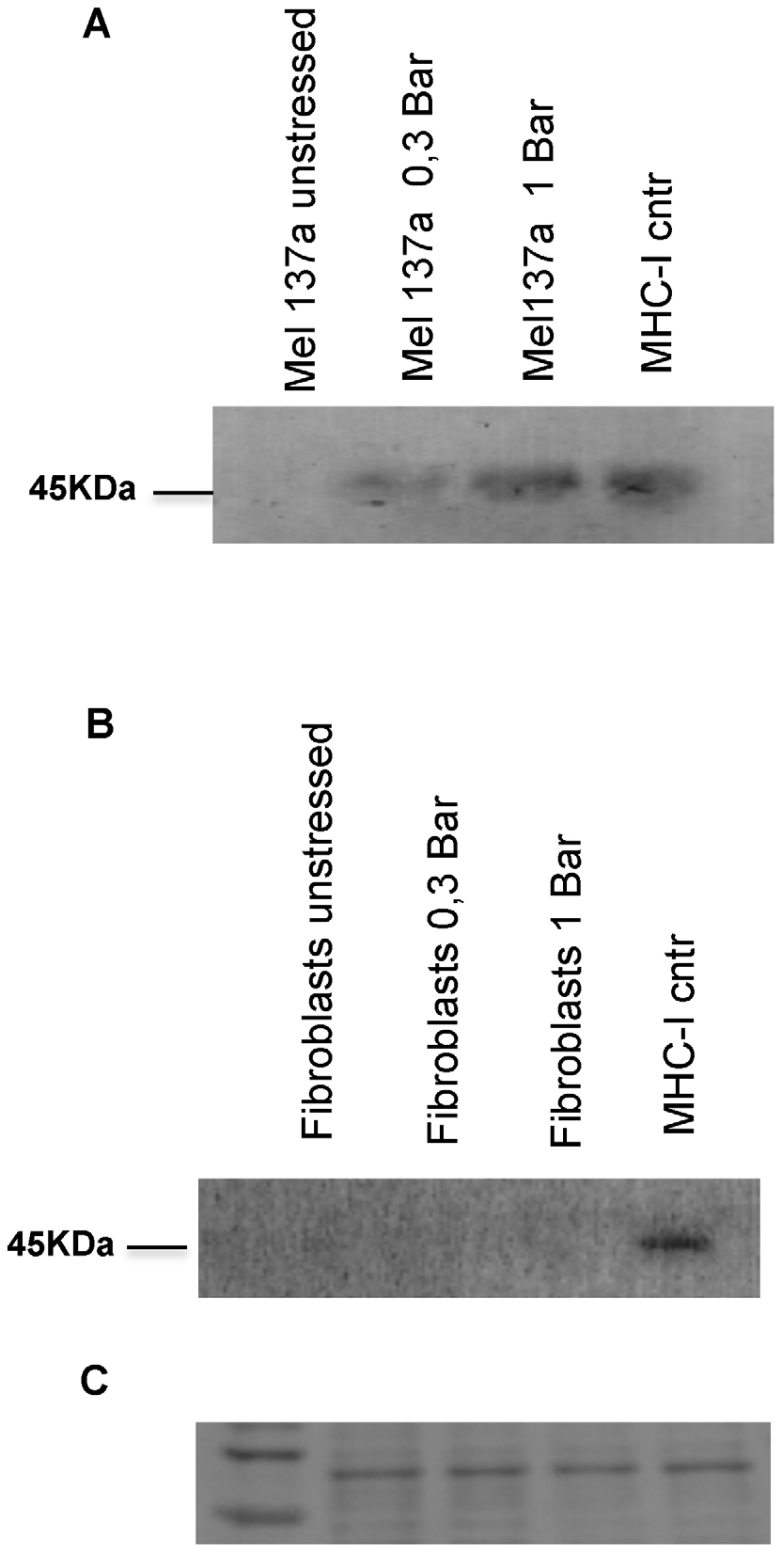
Western Blotting of MHC class I expression on the supernatants of treated samples: Tumor (A) and healthy cells (B) were analysed before and after mechanical stress by shock waves. MHC-I has molecular weight of 45 kDa. (C) Membrane incubated with Ponceau S red staining solution, as loading controls.

In a distinct set of experiments, we investigated the possibility that the mechanical stress could induce the shedding of MHC class I from the cell membrane. In the Fig. 7A and B, the Mel 137a and the fibroblasts cells were treated whit shock waves at 0.3 and 1 Bar of power. Supernatant from stressed cells and related controls were harvested. Their protein content was analysed by western blotting using an anti MHC class I monoclonal antibody as probe (Fig. 7A-B). The presence of bands at 0.3 and 1 Bar is correlated to the shedding of MHC-I from the cell membrane. Our data show that a high amount of MHC class I is lost from the tumor cell membrane upon mechanical stress.

## Discussion

Both devices used to stress cancer cells, i.e. the micropump and the shock waves [22], [39]–[40], were capable to specifically down-modulate the MHC class I. This effect is disease specific, since only tumor cells reduce their surface MHC class I levels upon mechanical stress stimulation. Moreover, we found that the controlled administration of stress increases the susceptibility of tumour cells to cytotoxic lymphocytes NK recognition.

Our data indicate that the mechanism by which mechanical stress induces the loss of MHC class I is not transcriptional but rather associated to the direct shedding of the molecule from the tumor cell membrane. We have indeed analysed the effect of mechanical stress on ATM/ATR signalling cascade [41] and on the SAPK/JNK pathway [42]. We found that mechanical stress did not affect the activation status of ATM, Chk1, Chk2, p53 and neither of JNK (S3 Fig.).

PCA analysis is found an effective statistical for biological samples such as proteins, cells, etc. [30], [38]. The results, shown in Fig. 3, on Raman spectra in the range of 700–1800 cm^−1^ discriminates the stress cell to control cells of each cell lines. In addition, the Raman analysis with the verification of flow cytometry findings regarding the reduction of MHC-I confirms the variation of α-helix is predominately from MHC-I in the spectral range of 1550–1750 cm^−1^.

The mechanical stress forces were titrated using 0.3, 0.6 and 1 bar and their effect on the down-regulation of MHC-I molecules and DNAM-1 ligands expression on cancer cells was analysed (S4A-C Fig). While a clear dose dependent effect on MHC class I down-regulation was found (S4A Fig.), no changes in DNAM-1 ligands expression were observed (S4C Fig.).

However, it is intriguing to observe that, in both experimental approaches, 1 Bar was the optimal pressure (force/surface) intensity to obtain the decrease of MHC class I expression and the enhancement of the NK cells recognition. A clear increase in the amount of shedding MHC class I soluble form was measured in the stressed cells supernatants, where 1 Bar pressure was the most effective. Western blotting analysis of the supernatant (S4B Fig.) shows a reciprocal effect of the mechanical stress forces. It remains to be addressed whether the physiologic forces associated with the human blood pressure ejection from the aortic arch may affect the MHC class I membrane expression of metastatic tumor cells during their hematological spreading with the mechanism above described. Mechanical stress switches the tumor phenotype from being NK resistant to NK susceptible. Our findings indicate an immunologically relevant effect of mechanical stress on the tumor susceptibility to lymphocytotoxic attack. We incidentally observed that the different behaviour in MHC class I shedding between healthy and cancer cell could be correlated with their different mechanical rigidity. In fact, as well known and measured in optical stretchers, cancer cells systematically show a higher deformability under mechanical forces [6]. The poorer rigidity of cancer cell, due to cytoskeleton reshuffling [43], induces a higher local membrane deformation that increases the detachment and the shedding of MHC class I. In our vision, this mechanism is responsible for the increased concentration of MHC class I in the supernatant.

Several reports indicate that tumorigenesis is mainly associated with changes in the phospholipids and protein content on biological membranes [44]–[47]. The data reported here provide further support to these observations, highlighting the distinct physical and chemical properties of cancer cell membranes compared to the normal ones and directly relate this observation with the cell immunogenicity. Moreover, it is possible to speculate that MHC class I molecules could differ for their biological properties (surface life span) accordingly with the chemical physical feature of the cell membrane lipid bilayers where they are expressed.

We further speculate that organs such as heart, and related tissues such as muscles, that posses mechanical activity in their normal function, and could generate mechanical stress, show a minor or absent presence of tumours [48]–[52]. The inherent mechano-kinetic activity could generate a self-healing mechanism as described above. In the future we are planning to further investigate along this direction.

We finally point out here that the use of ultrasound is particularly interesting for therapy treatments, due to their intrinsic macroscopic penetration depth (several centimetres) in human and animal tissues.

## Supporting Information

S1 Fig **Experimental set up for mechanical stress of tumor cells.** 1A: on the left, graphical representation of the mechanism for treating the cells by stressing them in between the gears of the micropump; left-bottom: SEM image of the micropump; on the right, scheme of the set-up used for treating the cells: A-micropump, B-motor activating the magnets inside the micropump allowing the gears to rotate; C-sample reservoir; E-sample inlet; F-Sample outlet. 1B: The instrument is equipped with a handpiece high-energy A and C, a manometer for operating pressure, an operating pressure control and a handpiece connection, B. Cell lines were treated in liquid, PBS or complete Medium, in petri dish, C.
(TIFF)

S2 Fig **Immunophenotypic analysis of 293T and IM9 cell lines under mechanical stress condition.** Expression on 293T of MICA, MICB, ULBP1–4, and the free heavy chain of MHC class I (A) and PVR and Nectin-2 on IM9 (B) were compared between stressed and not stressed cells. Data were expressed as fold decrease respect to the control, set as 1. In brief, each sample value was divided against the average of the control values. The so obtained data were used in statistical analysis. Statistical significance was measured used Mann – Whitney test.
(TIFF)

S3 Fig **Effect of mechanical stress on ATM/ATR signalling cascade and on stress-activated kinase.** Western blot of total extract from 293 T cells mechanically stressed or not for 1 h; the treatment with the damaging agent etoposide 5 µM for 1 h is shown as positive control; phospho-ATM, pChk1, pChk2, p53 and pJNK were analysed and MCM7 was used as loading control.
(TIFF)

S4 Fig **Mechanical stress effect on IM9 cell line immunophenotype.** A) Decrease at 0.3 and 0.6 Bar of MHC-I molecules expression on IM9 cell line. (n = 7 separate experiments, p = 0,0202; n = 7 separate experiments, p = 0,0010; p<0.05 respectively); B) MHC class I expression of IM9 cell supernatants was analysed with western blotting at different powers, compared to control cells. MHC-I has molecular weight of 45 kDa. C) No variation for PVR and Nectin-2 activator ligands after 0.3, 0.6 and 1Bar pressure treatments (n = 3 separate experiments). Statistical significance was measured used Mann – Whitney test.
(TIFF)
