# Correction: Mechanical Stress Downregulates MHC Class I Expression on Human Cancer Cell Membrane

**DOI:** 10.1371/journal.pone.0118519

**Published:** 2015-03-06

**Authors:** The PLOS ONE Staff

The fifth author’s first and last names were inadvertently switched. The first name appears as the last name and the last name appears as the first name. The correct name is: Tadepally Lakshmikanth. The correct citation is: La Rocca R, Tallerico R, Talib Hassan A, Das G, Lakshmikanth T, Matteucci M, et al. (2014) Mechanical Stress Downregulates MHC Class I Expression on Human Cancer Cell Membrane. PLoS ONE 9(12): e111758. doi:10.1371/journal.pone.0111758
