# Supplementary figures and images for: Mechanical Stress Downregulates MHC Class I Expression on Human Cancer Cell Membrane
Source: PLoS One. 2014 Dec 26;9(12):e111758. doi: 10.1371/journal.pone.0111758 (PMC4277281; doi:10.1371/journal.pone.0111758)

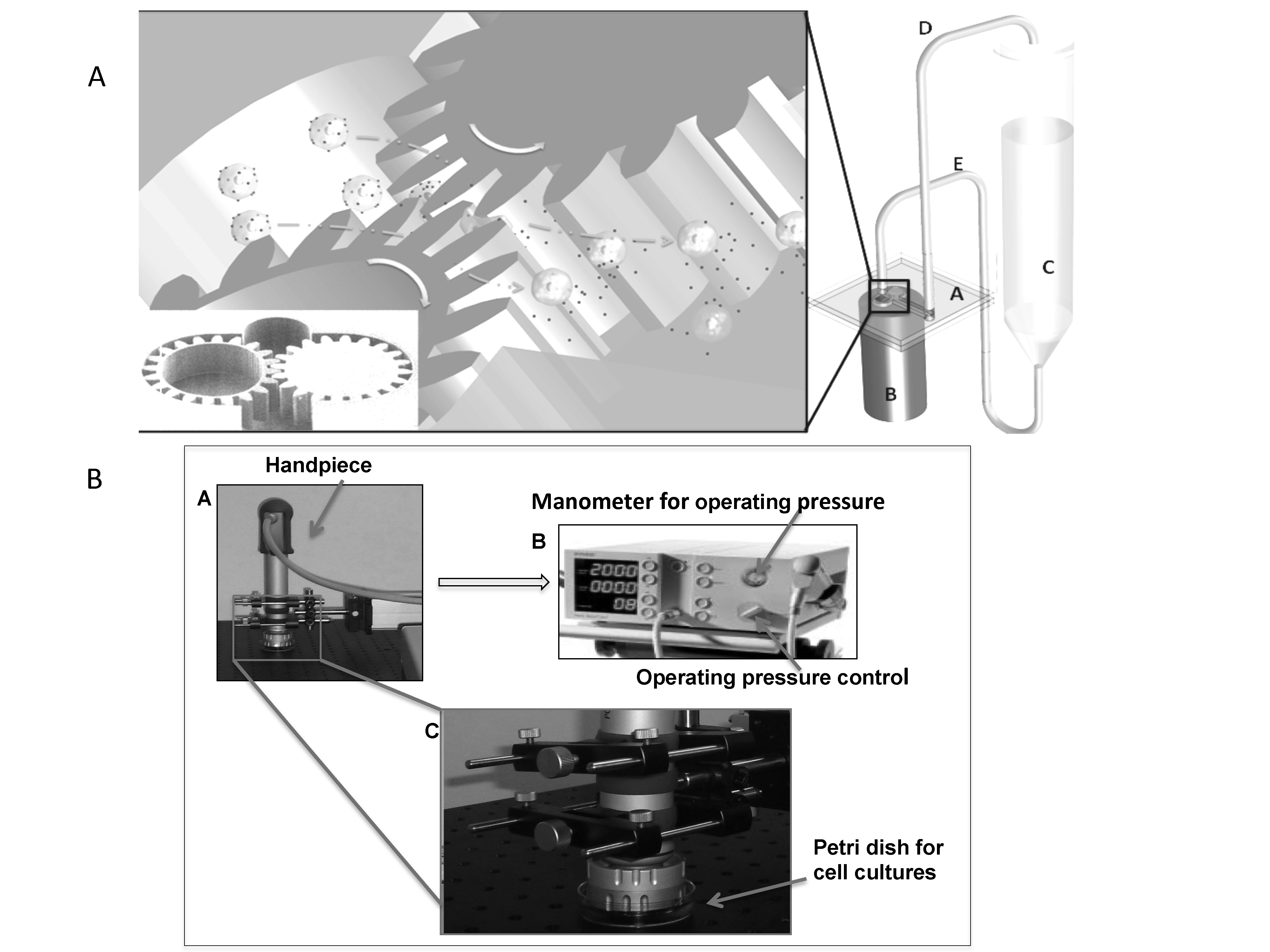

Supplement: S1 Fig — Experimental set up for mechanical stress of tumor cells. 1A: on the left, graphical representation of the mechanism for treating the cells by stressing them in between the gears of the micropump; left-bottom: SEM image of the micropump; on the right, scheme of the set-up used for treating the cells: A-micropump, B-motor activating the magnets inside the micropump allowing the gears to rotate; C-sample reservoir; E-sample inlet; F-Sample outlet. 1B: The instrument is equipped with a handpiece high-energy A and C, a manometer for operating pressure, an operating pressure control and a handpiece connection, B. Cell lines were treated in liquid, PBS or complete Medium, in petri dish, C. (TIFF) [file pone.0111758.s001.tiff]

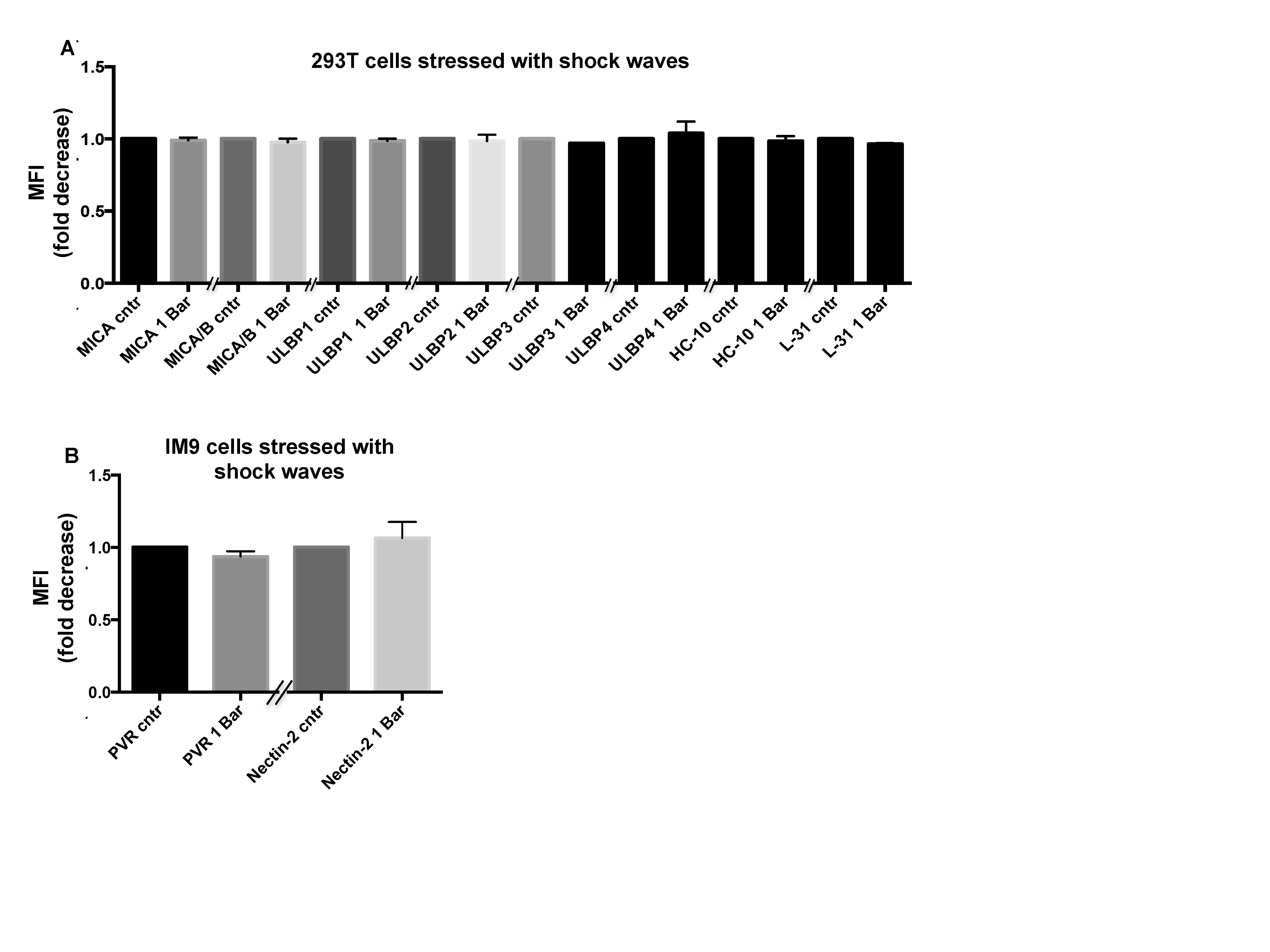

Supplement: S2 Fig — Immunophenotypic analysis of 293T and IM9 cell lines under mechanical stress condition. Expression on 293T of MICA, MICB, ULBP1–4, and the free heavy chain of MHC class I (A) and PVR and Nectin-2 on IM9 (B) were compared between stressed and not stressed cells. Data were expressed as fold decrease respect to the control, set as 1. In brief, each sample value was divided against the average of the control values. The so obtained data were used in statistical analysis. Statistical significance was measured used Mann – Whitney test. (TIFF) [file pone.0111758.s002.tiff]

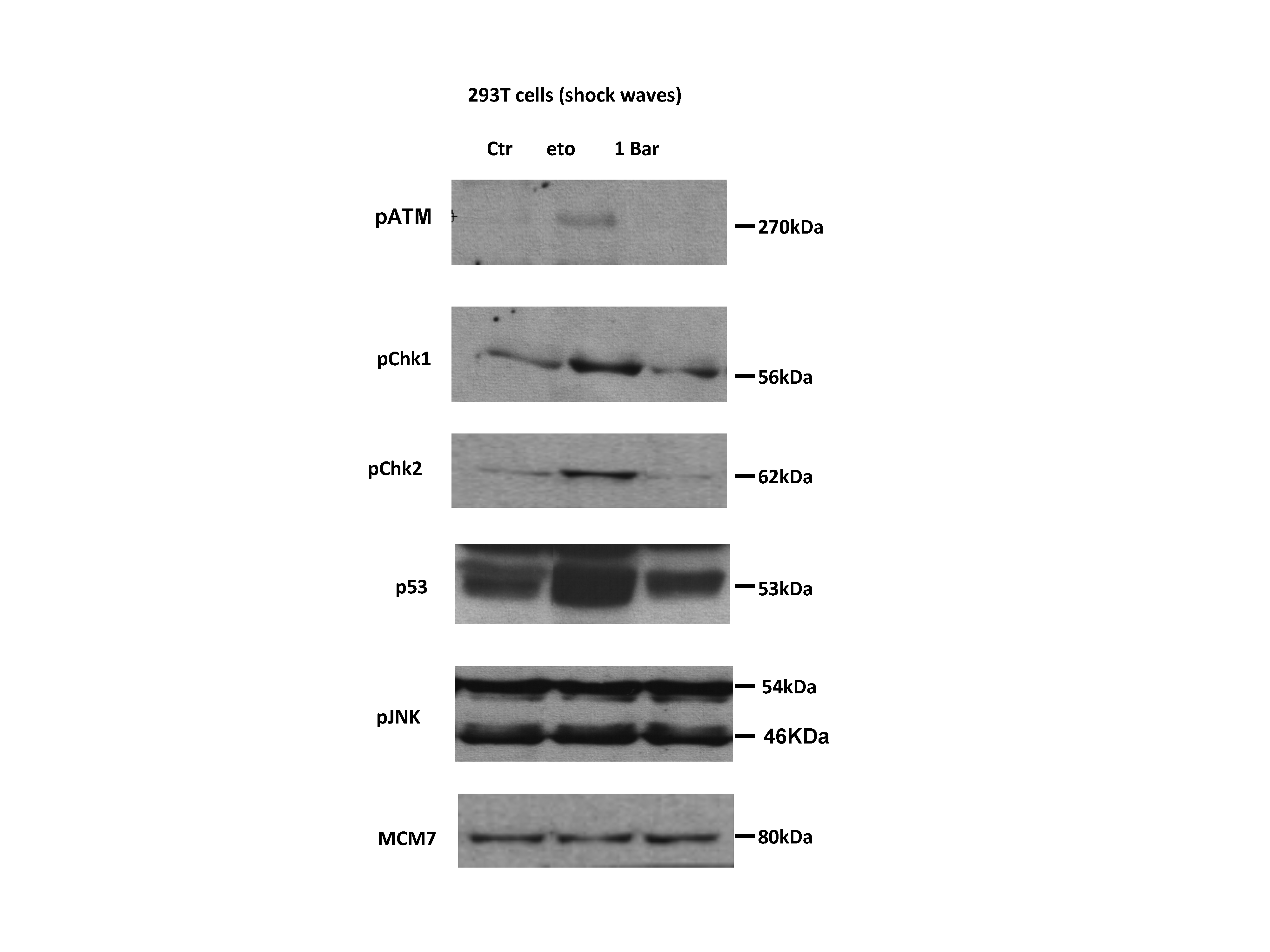

Supplement: S3 Fig — Effect of mechanical stress on ATM/ATR signalling cascade and on stress-activated kinase. Western blot of total extract from 293 T cells mechanically stressed or not for 1 h; the treatment with the damaging agent etoposide 5 µM for 1 h is shown as positive control; phospho-ATM, pChk1, pChk2, p53 and pJNK were analysed and MCM7 was used as loading control. (TIFF) [file pone.0111758.s003.tiff]

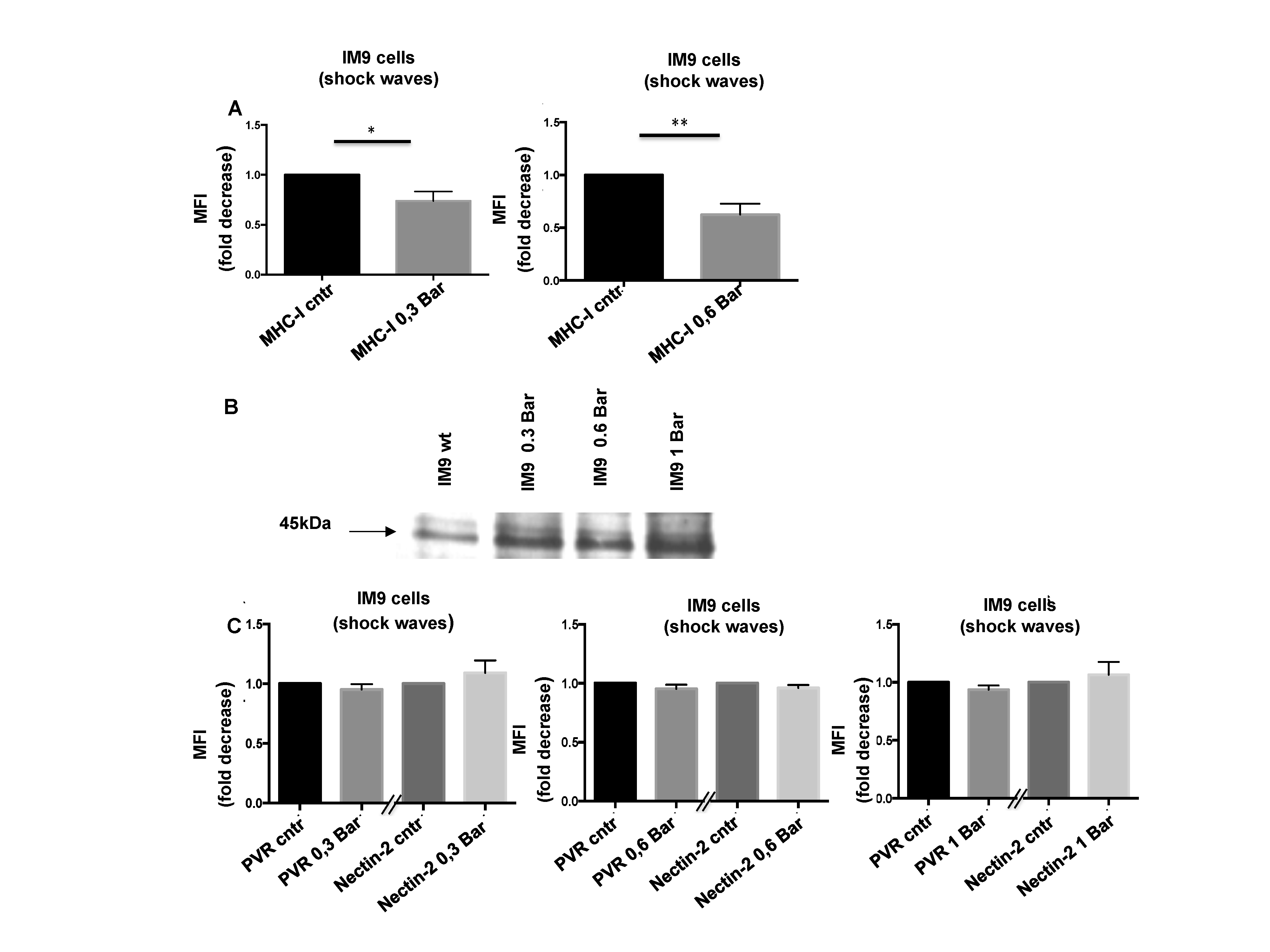

Supplement: S4 Fig — Mechanical stress effect on IM9 cell line immunophenotype. A) Decrease at 0.3 and 0.6 Bar of MHC-I molecules expression on IM9 cell line. (n = 7 separate experiments, p = 0,0202; n = 7 separate experiments, p = 0,0010; p<0.05 respectively); B) MHC class I expression of IM9 cell supernatants was analysed with western blotting at different powers, compared to control cells. MHC-I has molecular weight of 45 kDa. C) No variation for PVR and Nectin-2 activator ligands after 0.3, 0.6 and 1Bar pressure treatments (n = 3 separate experiments). Statistical significance was measured used Mann – Whitney test. (TIFF) [file pone.0111758.s004.tiff]
